# Parental Self-Efficacy—A Predictor of Children's Health Behaviors? Its Impact on Children's Physical Activity and Screen Media Use and Potential Interaction Effect Within a Health Promotion Program

**DOI:** 10.3389/fpsyg.2021.712796

**Published:** 2021-08-12

**Authors:** Katrin Kieslinger, Olivia Wartha, Olga Pollatos, Jürgen M. Steinacker, Susanne Kobel

**Affiliations:** ^1^Department Clinical and Health Psychology, Institute of Psychology and Education, Ulm University, Ulm, Germany; ^2^Division of Sports and Rehabilitation Medicine, Centre of Medicine, Ulm University Hospital, Ulm, Germany

**Keywords:** parental self-efficacy, physical activity, parents, media use, screen time, prevention, kindergarten children

## Abstract

Insufficient physical activity (PA) and increased screen media use (SMU) can have detrimental effects on children's health. Parental self-efficacy (PSE) can act as an important predictor for a healthy upbringing. The aim of this study was to investigate the influence of PSE on children's PA and SMU. Additionally, a moderating effect of PSE on the intervention effect of the health promotion program “Join the Healthy Boat” was examined. Using a prospective randomized controlled trial, 558 kindergarten children (3.6 years [*SD* = 0.6]) were examined. Data was collected using parental self-report. A significant influence of PSE on children's PA (*B* = 0.33, *p* = 0.025) and children's SMU (*B* = 0.42, *p* = 0.006) was found. The moderating effect of PSE on the intervention effect was neither significantly related to children's PA (*p* = 0.360) nor to children's SMU (*p* = 0.531). This confirms the importance of PSE on children's health development. Despite the lack of a moderating effect, interventions should also promote healthy activity behaviors and self-efficacy for parents in order to engage children in a healthy lifestyle.

## Introduction

High prevalence of overweight and obesity pose a worldwide health risk for children and adolescents and subsequently across their lifespan (Skinner et al., 2015; Abarca-Gómez et al., 2017; GBD 2015 Obesity Collaborators, 2017; Sagar and Gupta, 2018; Garrido-Miguel et al., 2019). In 1975, worldwide, five million girls and six million boys aged 5–18 were overweight (Abarca-Gómez et al., 2017). In 2016, 41 years later, more than 50 million girls and 74 million boys reached an overweight or obese status (Abarca-Gómez et al., 2017). A prospective study showed that 90% of children who were obese at an age of 3 years were also overweight or obese as adolescents (Geserick et al., 2018). Furthermore, a simulation showed that obesity in adulthood can be predicted by an early development of obesity (Ward et al., 2017). This highlights the importance of taking preventive measures against obesity, particularly in early years, since childhood overweight and obesity increase the likelihood of developing other diseases such as diabetes, coronary heart disease or cancer in adulthood (Llewellyn et al., 2016).

Promoting healthy physical activity and screen media use is one way to reduce the number of overweight children (Katzmarzyk et al., 2015; Ling et al., 2016; Kobes et al., 2018). Both the trend toward less physical activity and increasing digitalization already have an impact on children's health (Medienpädagogischer Forschungsverbund Südwest, 2014, 2018; Domingues-Montanari, 2017; Finger et al., 2018). Sufficient physical activity and time-controlled screen media use are important factors, which affect a healthy development of children into adulthood (Saunders and Vallance, 2017; Biddle et al., 2019).

The World Health Organization (WHO), which defines physical activity as any “movement of the body that uses energy over and above resting” recommends a minimum of 180 min of daily physical activity at any intensity with at least 60 min of moderate to very strenuous intensity for 3–4-year-olds (World Health Organization, 2019). In Germany, daily physical activity levels of at least 60 min are achieved by no more than 42.5% of girls and 48.9% of boys aged 3–6 years (Finger et al., 2018).

Another strong determinant of young children's health is sedentary behavior (Poitras et al., 2017). In times of digitalization and technological change, screen media use is becoming increasingly important in kindergarten children's leisure time (Hinkley et al., 2010; Radesky and Christakis, 2016; Canadian Paediatric Society, 2017). Recommendations on “sedentary screen time” defined as “time spent passively watching screen-based entertainment (TV, computer, mobile devices” (World Health Organization, 2019, p. 5) state that children aged 3–4 years should use screen media for no more than 1 h per day (World Health Organization, 2019). In Germany, 44% of 2–5-year-olds watch television daily in addition to video/DVD or computer/console/online games and the use of smartphones and tablets, resulting in an average of 43 min per day (Medienpädagogischer Forschungsverbund Südwest, 2014). Longitudinal investigations show that screen media habits such as heavy television use in childhood persist into early adulthood (York, 2016). Therefore, early childhood is an important period for implementing preventive measures to promote a healthy lifestyle including reduced screen media use in order to prevent potential subsequent developments detrimental to health (York, 2016; Canadian Paediatric Society, 2017).

For instance, there is a clear association between children's television viewing of more than 2 h per day and lower well-being, less self-control and emotional stability (Twenge and Campbell, 2018), decreased fitness (Hardy et al., 2018), lower school and academic performance (Howie et al., 2020; Mineshita et al., 2021), reduced social skills (Hinkley et al., 2010), cardio-vascular diseases (Lissak, 2018) as well as mental and social disorders (Pagani et al., 2016; Stiglic and Viner, 2019).

Yet, regular and sufficient physical activity is associated with favorable metabolic health (Oliveira and Guedes, 2016), healthy body composition and better cardiorespiratory fitness (Füssenich et al., 2015), positive effects on motor skills (Zeng et al., 2017), a reduced likelihood of psychological and cardiometabolic complaints and better cognitive and psychosocial skills (Carson et al., 2017; Biddle et al., 2019; Rodriguez-Ayllon et al., 2019).

Both of those health behaviors are known to be associated with parental education status as well as the family's socio-economic and migration background (Ball, 2015; Lepeleere et al., 2015; Finger et al., 2018). Parents play an important role in guiding their children's health behaviors in the early years (Wittkowski et al., 2016; Lee et al., 2018). One important component in behavior change is the psychological construct “self-efficacy” (SE) (Bandura, 1997). It is based on social cognitive (learning) theory (Bandura, 1997, 2001) and is defined as “people's beliefs about their capabilities to produce designated levels of performance that exercise influence over events that affect their lives” (Bandura, 1994, p. 2).

SE is dynamic and can be developed by any person (Schunk and Ertmer, 2000). Moreover, it is strongly related to one's own competence and future behavior. However, it is not the actual abilities that are important, but rather confidence in one's own abilities (Bandura, 1977). Solely with the knowledge that one's own abilities are sufficient to show the desired behavior, behavior modification is possible (Bandura, 1977).

General SE, which describes a general, optimistic appraisal of life coping skills, that includes all areas of life, is to be distinguished from SE related to a specific area (Bandura, 1997). Parental self-efficacy (PSE), a specific form of SE, describes the subjective conviction or belief in one's own abilities to be a good parent and influence the child in a way that promotes health and success (Jones and Prinz, 2005; Glatz and Buchanan, 2015; Benedetto and Ingrassia, 2018; Albanese et al., 2019). High PSE is associated with confidence in acquiring and practicing effective parenting skills. Conversely, parents with low PSE find it more difficult to solve challenging parenting situations effectively (Jones and Prinz, 2005). PSE influences parental competence as well as child development and thus contributes to the success of parenting (Jones and Prinz, 2005). Therefore, PSE is thought to have strong effects on child development and health (Albanese et al., 2019).

Due to the multiple ways in which PSE affects children, the construct is gaining increasing attention in child health promotion (Jones and Prinz, 2005; Ekim, 2016; Möhler et al., 2020). Especially physical activity and media use represent two behaviors that could be affected by PSE (Smith et al., 2010). A meta-analysis presented heterogeneous data regarding the relationship of PSE on children's physical activity and screen media use (Xu et al., 2015). Only an Australian cross-sectional study showed that preschool children whose parents reported higher PSE were less likely to undercut the physical activity recommendations of 3 h per day (Smith et al., 2010). Similarly, a Swedish study showed that maternal SE was associated with higher MVPA (moderate-to-vigorous physical activity) in their 4-year-old children. Whereas no correlations were seen for light forms of exercise or sedentary behavior (Rohde et al., 2018).

At ages 6 years old and younger, a clear relationship also emerged between PSE and children's screen media behavior (Xu et al., 2015). Preschool children of parents with lower PSE exceeded the recommendation of no more than 2 h of daily screen media use significantly more often than children of more self-efficacious parents. PSE is thus associated with children's physical activity and screen media behavior (Smith et al., 2010). However, studies have also shown contrary results and suggest a different impact of PSE on physical activity and screen media behavior depending on children's gender when aged 6–12 years (Lepeleere et al., 2015). Since most of those associations increase with age (Smith et al., 2010), early health promotion is vital to enhace children's chances for a healthy lifestyle.

This indicates that it is particularly important to identify predictors that increase physical activity behavior and reduce screen media use, thus positively influencing children's health. It has been shown that PSE is an important factor in influencing children's physical activity and screen media use positively. Despite this, some ambiguous relationships still emerge that need to be further examined and elucidated. To the best of our knowledge, there are no studies investigating the influence of PSE on physical activity and screen media use of German kindergarten children; and neither is there a study considering the effect of PSE in terms of success of an intervention. In order to uncover previously unexplored mechanisms of PSE, data of the health promotion program “Join the Healthy Boat” were examined and presented below.

“Join the Healthy Boat” is a health promoting program for (pre-)school children that tries to incorporate all of those aspects mentioned above: physical activity promotion, reduction of screen media use and involvement of parents in order to increase their PSE.

Aim of the current study was therefore to investigate the influence of PSE on kindergarten children's physical activity and screen media behavior. Additionally, it was tested how PSE changes the effect of the health promoting intervention “Join the Healthy Boat” on kindergarten children's physical activity and screen media behavior.

## Materials and Methods

### Intervention

The “Intervention Mapping Approach” (IMA) (Bartholomew et al., 2006) was used to design the intervention of “Join the Healthy Boat” (Wartha et al., 2016). Based on a need assessment, concrete program targets were specified. Using theoretical models, such as Bandura's socio-cognitive theory (Bandura, 2001) and Bronfenbrenner's socio-ecological approach (Bronfenbrenner, 1979), concrete applications for health promotion were established and conceptualized. The subsequent implementation in kindergartens and the program's evaluation were planned and carried out based on the above-mentioned framework (Wartha et al., 2016).

Similarly, to the intervention designed for primary schools (Wartha et al., 2017), the key topics of the interventions are increasing physical activity and meaningful leisure time activities including reduced screen media use. A healthy diet is targeted by increasing fruit and vegetable intake as well as reducing sugar sweetened beverage consumption. The materials, which consist of 20 exercise and games lessons as well as 30 ready-to-use-ideas of various lengths, were designed to be integrated into the daily life of participating kindergartens. Through healthy eating, physical activity and leisure time units, children can explore alternatives for their behavior, gain knowledge about their bodies and health as well as improve their motor skills, physical activity, and healthy eating habits. Additionally, short activity games, which can be used twice daily, were implemented. To also include parents, two parents' nights took place and parental letters were provided in three different languages (Kobel et al., 2017). In order to implement the program in kindergartens sustainably, kindergarten teachers were trained and received the bespoke materials, including instructional behavioral and educational resources free. They then implemented the program throughout 1 year in their kindergarten so children would experience a health-promoting environment, which would make it easier to enhance their health and well-being. On average, children received two units per week in addition to the two daily short activity games.

### Study Design and Participants

The study was designed as a prospective randomized controlled trial with two points of measurement.

All 7,937 kindergartens in South-west Germany were made aware of the intervention program and its evaluation study by mail. Sixty-six kindergartens showed interest in participating in the study. However, four of those had to be excluded due to staffing and organizational reasons. To ensure a similar number of participants between intervention and control group, kindergartens were stratified based on the number of kindergarten children per kindergarten (method of stratification in Kobel et al., 2017).

Initially, 1,012 children from 62 kindergartens planned to participate, equally distributed in the intervention and control groups. Due to further dropouts, 57 kindergartens, 30 in the intervention group and 27 in the control group, (*N* = 973 children) took part in baseline measurements.

After the baseline measurements were conducted, all parents received a questionnaire about their child's health and items that depict the family environment. Kindergarten teachers of the intervention group received their training and ready-to-use materials to implement the intervention in the kindergartens. Kindergarten teachers in the control group carried on as usual and received training and materials the year later, after follow-up measurements were completed in both, intervention and control group.

Therefore, for the present study, 558 kindergarten children from south-west Germany, aged 3–5 years (and later 4–6 years) were examined at two points of measurement and divided into 318 participants in the intervention group and 240 participants in the control group.

The study was registered at the German Register of Clinical Trials (DRKS; DRKS00010089). In addition, the study protocol has been approved by the Ethics Committee and the Ministry of Culture and Education and was carried out according to the Helsinki Declaration. Parents gave their written informed consent for the study, the children their assent on site.

### Measures

Data were collected objectively during a visit at the kindergarten, and subjectively by means of a questionnaire, the parents completed. Physical activity, screen media use and PSE were assessed at baseline and follow-up via a parental questionnaire. Children's physical activity was assessed by their parents using the following question: “On how many days of a normal week is your child physically active for a total of at least 60 min a day so he or she gets out of breath or begins to sweat?”. Parents were given a scale from 0 to 7 days of the week to answer the item. The included items for physical activity as well as screen media use were taken from a validated instrument used in a large German study (German Health Interview and Examination Survey for Children and Adolescents [KiGGS]) with 18,000 German children and adolescents (Lange et al., 2014) and validated against assessment by accelerometry (Kahlert and Brand, 2011). The cut-off of 60 min for determining sufficient physical activity was set on basis of WHO physical activity guidelines (World Health Organization, 2019).

To assess children's screen media behavior, children's average duration of screen media use on a normal day during the week and on the weekend was given by their parents. Parents responded to “On an average weekday, how much time does your child spend doing the following?” and “On an average Saturday/Sunday, how much time does your child spend with the following activities?” on a 7-point Likert scale, ranging from 0 “not at all” to 7 “more than 3 h” for watching TV/playing video games, using the computer and tablet computer or using a mobile phone/smartphone. For good comparison, the here used questions were also taken out of the KiGGS questionnaire (Lange et al., 2014). One hour was used as cut-off for determining high screen media use, since WHO recommends no more than 1 h per day (World Health Organization, 2019).

PSE was assessed using a self-assessment on a scale from “strongly disagree” to “strongly agree” (Likert-7) and was adapted from other validated scales (Bohman et al., 2014). With regard to their children's physical activity, parents were asked to rate whether they “manage to ensure that their child gets sufficient physical activity, even if.” for example, “the weather is bad.” In addition, PSE related to children's screen media use was assessed with the item “I manage to make sure my child doesn't watch as much TV, even if.” for example “one of his or her favorite shows comes.”

Socioeconomic status consisted of parents' highest educational and occupational qualification, parents' highest occupational status and net household income (Lampert et al., 2014). A migration background was defined as “children with at least one parent born in a foreign country or children who were spoken to in a foreign language for the first 3 years or their life” (Settelmeyer and Erbe, 2010; Federal Statistical Office, 2019). Anthropometric measurements were taken during a visit at kindergarten. Body weight and height were measured by trained staff according to ISAK standards (Marfell-Jones et al., 2012) using calibrated and tared body scales (Seca 826, Hamburg) and a portable stadiometer (Seca 217, Hamburg) in the morning.

National percentiles of BMI were used to determine children's weight status (Kromeyer-Hauschild et al., 2001). A child was considered overweight if their BMI percentiles ranged between the 90th and 97th percentile and obese if their percentiles were greater than 97. Further, children whose BMI percentiles were below the 10th percentile were classified as underweight (Kromeyer-Hauschild et al., 2001).

### Data Analysis

To detect significant differences between the two groups, non-parametric Mann–Whitney-*U*-tests were calculated. Due to the lack of normal distribution, Shapiro–Wilk-test was used to calculate descriptive values. A significance level of 5% and a confidence interval of 95% were chosen for all calculations.

In order to ensure groups of equal size and to be able to perform a more robust calculation, the dependent variable “children's physical activity” and “children's screen media use” were dichotomized, respectively. Due to the dichotomization (categorical dependent variable) and in order to calculate and solve multivariate problems, logistic regressions were chosen as the method for inferential statistical analyses. In the case of physical activity, a cut-off value of four days was chosen to ensure sufficient physical activity on most days of a week. To determine children's screen media use, the average time of screen media use during a usual week (including weekdays and weekends) was calculated and dichotomized at the cut-off of 1 h of screen media use per day (World Health Organization, 2019). For PSE (children's physical activity as well as their screen media use), the average of the six associated items was calculated. The assumption of a similar distribution of the items was checked in advance. All statistical analyses were performed using IBM SPSS Statistics 26 (SPSS Inc., Chicago, IL, US).

## Results

The sample included 558 kindergarten children, 318 (56.99%) in the intervention group, 240 in the control group. There were no significant differences with regards to age, height, weight, BMI percentiles, weight status, migration background and low socio-economic status between the two groups. Yet, there were significantly more boys in the intervention group, compared to the control group (Table 1).

**Table 1:** Sample description baseline (children with participation at baseline and follow-up).

**Characteristic**	**Missing values**	**Intervention**	**Control**	**Total**
		**(*N* = 318)**	**(*N* = 240)**	**(*N* = 558)**
Gender^*^ (boys [*n*; %])	0	179 (56.29)	113 (47.08)	292 (52.32)
Age (years [*M*; *SD*])	0	3.64 (0.58)	3.63 (0.54)	3.63 (0.56)
Height (cm [*M*; *SD*])	71	105.17 (5.73)	105.05 (5.54)	105.12 (5.65)
Weight (kg [*M*; *SD*])	77	17.42 (2.45)	17.19 (2.69)	17.32 (2.55)
BMI percentiles [*M*; *SD*]	77	51.90 (25.87)	47.91 (25.91)	50.27 (25.93)
Overweight (incl. obese)^a^ [*n*; %]	77	18 (6.30)	10 (5.10)	28 (5.80)
Migration background^b^ [*n*; %]	116	79 (31.90)	73 (37.60)	152 (33.40)
Low socio-economic status [*n*; %]	222	29 (15.50)	30 (20.10)	59 (17.56)

*M (SD): mean (standard deviation)*.

* *Significant difference between control and intervention group, X^2^_(1)_ = 4,285, p = 0.04*.

a *>90th BMI percentile (Kromeyer-Hauschild et al., 2001)*.

b *Children with at least one parent born in a foreign country or children who were spoken to in a foreign language for the first 3 years or their life*.

There were no significant changes between baseline and follow-up regarding children's physical activity in intervention or control group. In the intervention group, the proportion of children achieving the WHO physical activity recommendations (World Health Organization, 2019) on 4 days or more per week increased slightly from baseline to follow-up (30.2 vs. 31.1% for baseline and follow-up, respectively), whereas in the control group the number decreased slightly (26.2 vs. 22.6% for baseline and follow-up, respectively). Children in the intervention group were active for 2.72 (*SD* = 2.02) days per week at baseline and for 3.09 (*SD* = 2.11) days per week at follow-up, while in the control group children were active on 2.57 (*SD* = 2.03) and 2.50 (*SD* = 1.90) days per week, respectively.

Similarly, there were no significant changes in children's screen media use between baseline and follow-up, neither in intervention nor control group. In the intervention group 49.8% of children used screen media for <1 h per day at baseline; at follow-up this decreased to 43.7% of children. Similar changes were observed in the control group with 51.4% of children using screen media for <1 h per day at baseline and 48.8% of children at follow-up.

At baseline, PSE regarding children's physical activity differed significantly between intervention and control group [5.14 (*SD* = 1.45) and 4.83 (*SD* = 1.51) points out of seven possible points for intervention and control group, respectively; *U* = 23 176.00, *p* = 0.023, *r* = 0.11]. This remained virtually the same at follow-up with an average of 5.19 (*SD* = 1.38) in intervention and 4.95 (*SD* = 1.36) in control group (*U* = 16 744.00, *p* = 0.043, *r* = 0.10).

For PSE in relation to screen media behavior, similar values were found, but no significant group differences. At baseline, PSE regarding children's screen media use was 5.45 (*SD* = 1.45) and 5.34 (*SD* = 1.48) out of seven possible points for intervention and control group, respectively. At follow-up, this increased slightly to 5.36 (*SD* = 1.44) and 5.45 (*SD* = 1.43) for intervention and control group, respectively.

### Parental Self-Efficacy as Predictor of Children's Physical Activity

PSE at baseline was a significant predictor for children's physical activity at follow-up (*B* = 0.33, *p* = 0.025, Table 2). If PSE was increased by one unit at baseline, the odds of higher levels of children's physical activity increased by 39.00% at follow-up (95% *CI*: 1.04–1.86). If evaluated gender-specifically, the effect remained significant for girls only (girls: *B* = 0.82, *p* = 0.031; boys: *B* = 0.24, *p* = 0.186).

**Table 2:** Parental self-efficacy at baseline as predictor of children's physical activity at follow-up.

**Predictor**	***B***	***SE***	***p***	***Exp*** **(*B*)**	**95% CI for *Exp*(*B*)**
Constant	−5.47	1.82	0.003	0.04	
PSE (B)^a^	0.33	0.15	0.025	1.39	[1.04–1.86]
Child PA (B)^b^	1.85	0.40	0.000	6.35	[2.89–13.95]
Gender	−0.93	0.40	0.021	0.40	[0.18–0.87]
Age	0.53	0.34	0.124	1.69	[0.87–3.31]
BMI-Percentiles	0.00	0.01	0.252	1.01	[0.99–1.02]
Migration background^c^	−0.08	0.41	0.846	0.92	[0.41–2.06]
SES^d^	0.04	0.33	0.897	0.00	[0.55–1.98]

*X^2^_(7)_ = 40.32, p < 0.001, Model fit: Cox and Snell R^2^ = 0.18, Nagelkerke's R^2^ = 0.27*.

a *Parental self-efficacy at baseline*.

b *Children's physical activity at baseline*.

c *Children with at least one parent born in a foreign country or children who were spoken to in a foreign language for the first 3 years or their life*.

d *Socio-economic status (parents' highest educational/occupational qualification, parents' highest occupational status and net household income)*.

The final regression model also revealed children's physical activity at baseline and their gender as significant variables on children's physical activity at follow-up (Table 2).

### Parental Self-Efficacy as Moderator Between the Intervention “Join the Healthy Boat” and Children's Physical Activity

**Figure 1:**
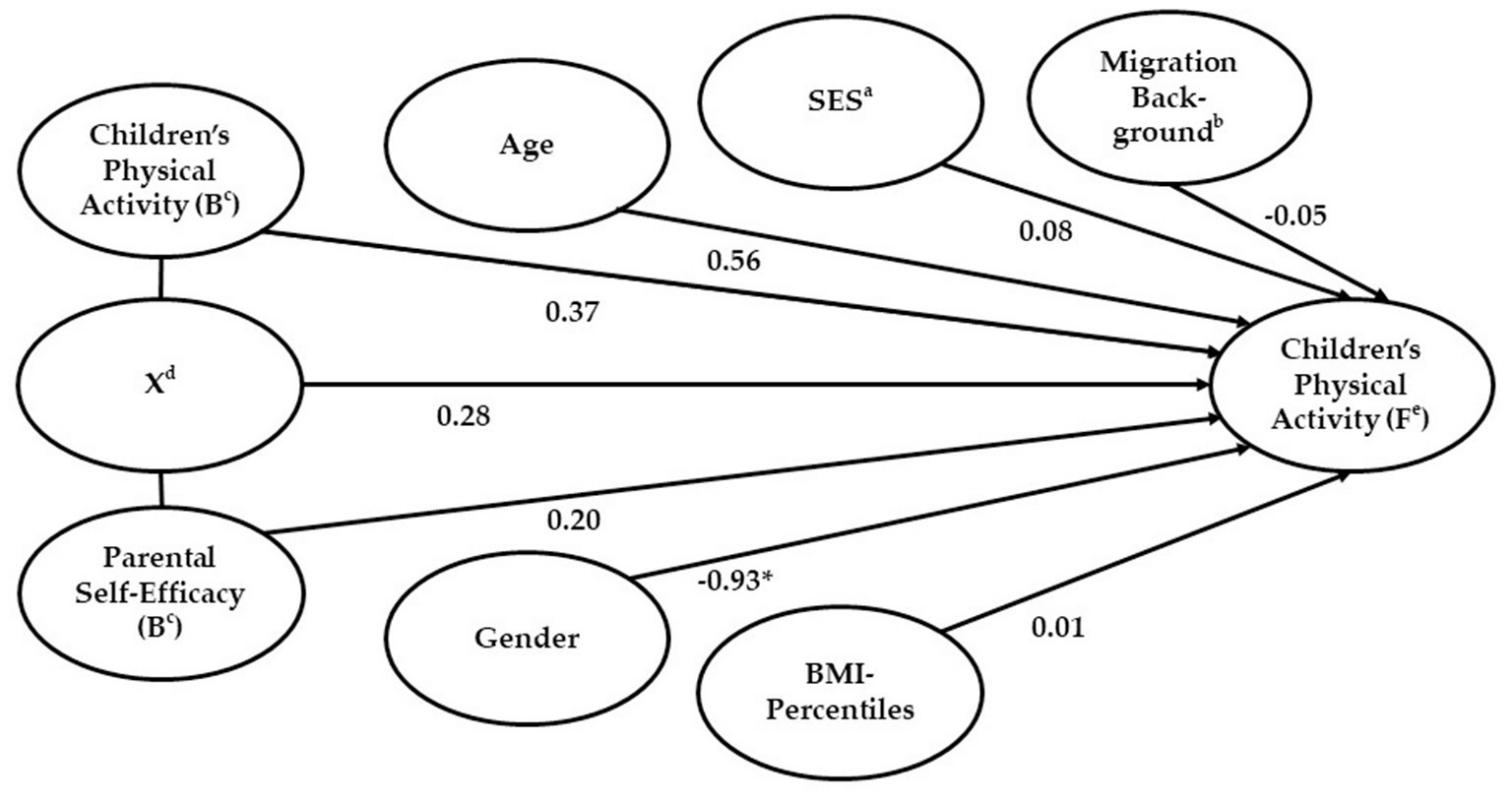
Parental self-efficacy at baseline as predictor of the intervention effect on children's physical activity. Results of the final model with all predictors (203 cases included [*X*^2^_(8)_ = 41.17, *p* < 0.001, Model fit: Cox and Snell *R*^2^ = 0.18, Nagelkerke's *R*^2^ = 0.28). Statistics are standardized regression coefficients. Lines represent relations. *p < 0.05. ^a^Socio-economic status (parents' highest educational/occupational qualification, parents' highest occupational status and net household income). ^b^Children with at least one parent born in a foreign country or children who were spoken to in a foreign language for the first 3 years or their life. ^c^B, baseline. ^d^X, interaction effect. ^e^F, follow-up.

Gender was found to have a significant effect on children's physical activity at follow-up, but no interaction effect of PSE at baseline and intervention effect (change of the children's physical activity from baseline to follow-up) could be found (Figure 1).

### Parental Self-Efficacy as Predictor of Children's Screen Media Use

PSE at baseline was a significant predictor of children's screen media use at follow-up (*B* = 0.42, *p* = 0.006; Table 3). If PSE at baseline was increased by one unit, the odds for greater screen media use of their children at follow-up increased by 52.9% (95% *CI*: 1.13–2.07). However, if evaluated gender-specifically, the effect was significant for girls only (girls: *B* = 0.98, *p* = 0.001; boys: *B* = 0.10, *p* = 0.619). Children's screen media use at baseline also predicted their screen media use at follow-up strongly and significantly (Table 3).

**Table 3:** Parental self-efficacy at baseline as predictor of children's screen media use at follow-up.

**Predictor**	***B***	***SE***	***p***	***Exp*** **(*B*)**	**95% CI for *Exp*(*B*)**
Constant	−2.94	1.81	0.105	0.05	
PSE (B)^a^	0.42	0.16	0.006	1.53	[1.13–2.07]
Screen media use (B)	2.29	0.40	0.000	9.90	[4.53–21.63]
Gender	0.54	0.36	0.138	1.71	[0.84–3.51]
Age	−0.29	0.33	0.373	0.75	[0.39–1.42]
BMI-Percentiles	0.01	0.01	0.080	1.01	[1.00–1.03]
Migration background^b^	−0.01	0.39	0.990	1.00	[0.46–2.16]
SES^c^	−0.19	0.33	0.571	0.83	[0.43–1.59]

*X^2^_(7)_ = 69.17, p < 0.001, Cox and Snell R^2^ = 0.31, Nagelkerke's R^2^ = 0.41*.

a *Parental self-efficacy at baseline (B)*.

b *Children with at least one parent born in a foreign country or children who were spoken to in a foreign language for the first 3 years or their life*.

c *Socio-economic status (parents' highest educational/occupational qualification, parents' highest occupational status and net household income)*.

### Parental Self-Efficacy as Moderator Between the Intervention “Join the Healthy Boat” and Children's Screen Media Use

**Figure 2:**
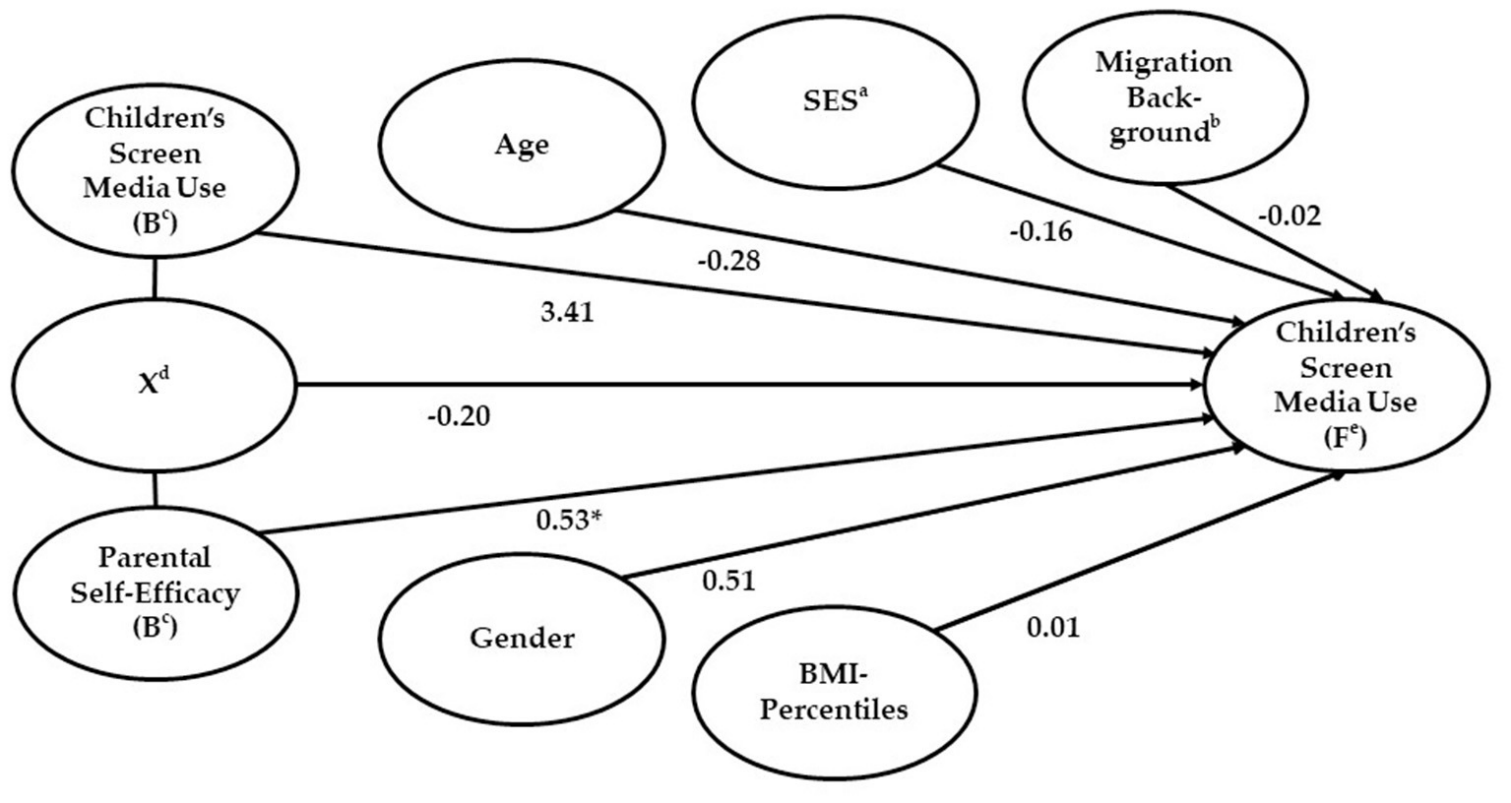
Parental self-efficacy as moderator between the intervention “Join the Healthy Boat” and children's screen media use. Results of the final model with all predictors (190 cases included; *X*^2^_(8)_ = 69.57, *p* < 0.001, model fit: Cox and Snell R*X*^2^ = 0.31, Nagelkerke's *R*^2^ = 0.41). Statistics are standardized regression coefficients. Lines represent relations. *p < 0.05. ^a^Socio-economic status (parents' highest educational/occupational qualification, parents' highest occupational status, and net household income). ^b^Children with at least one parent born in a foreign country or children who were spoken to in a foreign language for the first 3 years or their life. ^c^B, baseline. ^d^X, interaction effect. ^e^F, follow-up.

The model as a whole including all predictors was significant [*X*^2^_(8)_ = 69.57, *p* < 0.001, *n* = 190, Figure 2), but no significant interaction effect (X in Figure 2) could be observed. Therefore, even for children with more self-efficacious parents, the intervention effect (reduction of child's screen media use from baseline to follow-up) is not enhanced. PSE at baseline showed a significant effect on children's screen media use at follow-up. If PSE at baseline was increased by one unit, the probability that children used screen media for <1 h per day at follow-up increased by 79.6% (95% *CI*: 1.08–2.67; Figure 2).

## Discussion

This study investigated the influence of parental self-efficacy on children's physical activity and media behavior in a statewide sample of kindergarten children in Germany within 1 year in intervention and control group of a health promoting intervention. In addition, it was assessed whether PSE would change the effect of the intervention of the health promotion program “Join the Healthy Boat” on children's physical activity and children's screen media use. The results of PSE as a construct that can possibly enhance children's physical activity behaviors showed a significant positive effect of PSE on children's physical activity as well as on children's screen media behavior, especially that of girls.

### Parental Self-Efficacy as Predictor of Children's Physical Activity

The here found significant influence of PSE related to physical activity of kindergarten children is partly consistent with previous literature, which showed that toddlers of more self-efficacious parents moved significantly more in their daily lives than children of parents with lower SE (Bohman et al., 2014, Smith et al., 2010, Xu et al., 2015). However, there are also studies which did not report such associations (Heerman et al., 2017, Parekh et al., 2018). Yet, in those studies, different statistical analysis methods were used and children's physical activity was assessed objectively. Since in this study children's physical activity was recorded by parental reports and their socio-economic backgrounds differed significantly from those of the before mentioned sample (Heerman et al., 2017), these could be reasons for the different outcomes.

However, if analyzed with a gender context, it was seen that primarily girls benefit from PSE with regards to their physical activity. The significant positive effect of PSE on children's physical activity was lost for boys but strong for girls. It is well-known that boys are generally more physically active (Foweather et al., 2015; Nilsen et al., 2019), but these results suggest that parents, especially those with high PSE, have great potential to affect their girls' physical activity behaviors.

Apart from PSE, children's physical activity behavior at baseline was a significant predictor for their activity behavior 1 year later—independent of control or intervention group and PSE. Therefore, this was adjusted for in all analyses in order to control for a possible intervention effect.

Interventions have been known to affect results with PSE effects disappearing after controlling for intervention effects (Xu et al., 2014). In the present study, even after controlling for a possible intervention effect, a significant effect of PSE on children's physical activity behavior was recorded. Again, assessment methods vary between studies, for instance the selected self-efficacy scale. Xu et al. (2014) for example used items of global PSE in order to assess the effect of PSE on children's physical activity; in this study, specific ones addressing different aspects and situations of physical activity were used. According to Bandura (2006), SE can vary greatly between different domains. Therefore, a lack of specificity could potentially account for missing effects. Further, in order to assess PSE (in contrast to maternal SE or paternal SE) this study assessed PSE by questioning mothers and fathers about the same situations and physical activity aspects whereas other studies focused primarily on maternal SE and therefore assessed mothers' SE (Xu et al., 2014), which may lead to the difference in outcomes.

### PSE as Moderator Between the Intervention and Children's Physical Activity

In the present study, PSE was found to be a significantly positive predictor of children's physical activity at follow-up. Prior to the intervention, it was assumed PSE would have a moderating effect on health promoting aspects, such as physical activity promotion, reduction of sedentary time and screen media use, and therefore on its intervention effects. However, no moderating effect of PSE related to the intervention effect of “Join the Healthy Boat” on children's physical activity was found after 1 year of intervention. Therefore, although children's physical activity was significantly affected by PSE. The increase in physical activity from baseline to follow-up was more likely due to the intervention's key topics and materials rather than being moderated by PSE. PSE was hoped to be affected by introducing parental letters, parents' nights and so-called family homework, which all offered action alternatives in order to increase PSE and offer ideas and choices to increase children's physical activity.

### Parental Self-Efficacy as Predictor of Children's Screen Media Use

Not only was PSE a significant positive predictor for children's physical activity behavior; in this study, it was also significantly associated to children's screen media use. Similar results have been shown previously when investigating PSE and children's screen media behavior (Smith et al., 2010; Jago et al., 2015; Downing et al., 2017; Paudel et al., 2017). Yet, depending on age group and analyses, different outcomes have been reported. In 2-year-olds no association of PSE and children's screen media was found (Xu et al., 2014); again, possibly due to the assessment of global PSE among mothers instead of specific PSE related to children's screen media behavior reported by both parents as in this study. It was suggested that specific parental PSE related to children's screen media behavior would produce stronger associations on the reduction of screen time among kindergarten children (Bandura, 2006).

In the context of children's screen media use, their socioeconomic background as well as parental weight status have been shown to be associated to children's screen media behavior. Previous research showed significant effects of PSE on children's screen media use only among normal-weight parents of young children and those of moderate to high socio-economic status (Lepeleere et al., 2015). In the present study, those effects could be confirmed for the same age group and was controlled for the socio-economic status (as well as migration background). Parental weight status was not considered here. Possibly, the role of parental weight status should be included in future studies to further investigate its influence and parental perception on children's (health) behaviors.

As seen for children's physical activity, in this study, PSE was primarily a positive predictor of screen media behaviors in girls. This is something not been observed or at least not been reported previously for preschoolers (Lepeleere et al., 2015; Downing et al., 2017); for girls in grade 6 to 10 a significant difference in PSE on screen media use was seen before (Smith et al., 2010). However, this seems to suggest, that gender-specific different effects in younger children should be researched in more depth in future.

### PSE as Moderator Between the Intervention and Children's Screen Media Use

Despite the confirmed predicting effect of PSE on screen media use of kindergarten children in this study and similarly to the analyzed moderating effect of PSE on the intervention “Join the Healthy Boat” on children's physical activity, no moderating effect of PSE related to the intervention effect on children's screen media use was found after 1 year of intervention. Within the intervention, screen media reduction was addressed by lessons delivered by teachers including behavioral contracting and budgeting of screen media use, offering action alternatives for a screen-free leisure time as well as involving and informing parents through parental letters, parents' nights and family homework. Therefore, an attempt to increase PSE in specific “screen media domains” was made but screen media use at follow-up was largely predicted by children's screen media use at baseline and increased slightly within 1 year in control and intervention group (data not shown; Kobel et al., 2019). Possibly, the use of a health information website would have been more helpful or effective. In a previous study, 69% of parents reported to feel more self-efficacious in reducing at least two risk factors for obesity after using the “Healthy Families, Healthy Kids 2–5” website (Davies et al., 2014). Alternatively, the engagement in a group-based intervention could have helped, which was shown in a systematic review (Wittkowski et al., 2016). Studies that used task-specific measures of PSE recorded medium to large post-intervention effect sizes. In contrast, general measures of PSE just observed small to medium effect sizes (Wittkowski et al., 2016). Thus, a gap between recommended health behaviors (World Health Organization, 2019) and parents' actual knowledge could be closed and, in the process, PSE could be strengthened (Wittkowski et al., 2016).

Interventions should generally alter parental cognition, including PSE. This can be achieved by increasing knowledge and skills in a specific area, such as structured physical activity for (kindergarten) children. In the process, changes in the family and children's home environment most likely occur and should lead to healthier behaviors in families' daily lives (Mollborn and Lawrence, 2018). Therefore, the setting kindergarten is particularly suited for health promotion in young children. In “Join the Healthy Boat” parents are involved in the program and can directly transfer the learned content and behavioral changes to the family and child's everyday life. Other programs, such as an eight-week child-obesity intervention (Health Exercise Nutrition for the Really Young [HENRY]) demonstrated increases in PSE, in addition to increased physical activity and reduced screen time for children (Willis et al., 2014; Bridge et al., 2019).

PSE is emerging as a relevant factor that should also receive more attention from policymakers as well as health providers and health insurers. An increased offer of trainings in this area should be promoted especially with low-threshold possibilities. In order to save resources and reach parents as widely as possible, online interventions are a good complement to face-to-face services. Notwithstanding, it should be taken into account that research on PSE related to children's physical activity and screen media behavior still has gaps. Due to the partially heterogeneous data, the influence of PSE on children's physical activity and screen media behavior should be further elucidated. Nevertheless, the results of this study show clear directions for future research. In addition, the analysis of the effect of PSE on the intervention effect turned out to be novel. Future studies should examine a possible interacting effect, determining factors that contribute to this interaction.

### Strengths and Limitations

However, no study is without limitations, therefore, the following aspects should be considered when interpreting the here described findings. First, subjective assessment with its known biases: children's physical activity and screen media behavior were assessed using validated questions included in the German Health Interview and Examination Survey for Children and Adolescents (KiGGS), which previously assessed health behavior in 18,000 German children and adolescents (Lange et al., 2014). However, both behaviors were assessed subjectively and therefore amongst others prone to recall- and social desirability bias. Second, validation of instruments: for the assessment of PSE, items adapted from Bohman et al. (2014) were used but not validated in prior to its use. Since the completion of this study, some more scales to assess PSE have been developed (e.g., Bohman et al., 2016; Norman et al., 2018, Norman et al., 2021) using very similar items, which were designed according to scientific standards and based on Bandura's recommendations (Bandura, 2006). Third, a potential selection bias: participation was voluntary and it should be kept in mind that this study was conducted mainly on Caucasian, educated, and non-single parents and their children. Therefore, the results offer non-generalizable conclusions, which should not be applied without restriction to all cultures and socioeconomic groups. Forth, variables not considered: although results were adjusted for many co-variables, some—such as parental weight status—were not included, even though an association would have been possible (Lepeleere et al., 2015).

On the other hand, strengths of this study are the relatively large sample size, which may increase the likelihood of having sufficient power to detect intervention effects and its design as a prospective randomized control trail in a relatively wide area, including urban and rural areas. Next, the selected and used guidelines allow for international comparability and transparency. Lastly, PSE was assessed for specific domains by both parents, which permits an extensive in-depth view on PSE for children's physical activity and screen media behaviors.

## Conclusion

The results of this study confirm the positive influence of PSE on children's physical activity and screen media behavior, which emphasizes the importance of the construct for the implementation of health promotion and the preventive benefit for children. This study stands out as, to the best of our knowledge, it is the first to investigate the influence of PSE on physical activity and screen media behavior of kindergarten children based on a German sample.

Physical activity and screen media behavior are important determinants of healthy childhood development and should therefore be promoted from an early age (Katzmarzyk et al., 2015). Health consequences could be partially avoided by predictors that reinforce physical activity as well as reduce media use. If parents feel confident that they can positively influence their children's physical activity and screen media behaviors, their children may exhibit more physical activity and spend less time in front of screens. This insight provides an important basis for future societal, social, and political decisions.

Since a person's SE is not a stable, unchanging personality trait, but dynamic and variable due to situational or individual factors (Bandura, 1997), PSE should be specifically promoted, trained and, if necessary, modified in different settings. In this regard, it is important to educate parents and make them aware of the importance of PSE, preferably before their child is born. PSE is critically involved in the success of parenting. In addition, more self-efficacious parents influence child development more positively (Jones and Prinz, 2005).

There is also an opportunity to provide targeted interventions to improve PSE. This could be elements in larger health promotion programs or as a stand-alone intervention (e.g., Miller-Heyl et al., 1998; Wittkowski et al., 2016; Benedetto and Ingrassia, 2018). Already, some studies specifically confirm the effectiveness of interventions to promote PSE regarding children's physical activity and media behaviors (Jones and Prinz, 2005; Lepeleere et al., 2017).

## Data Availability Statement

The raw data supporting the conclusions of this article will be made available by the authors, without undue reservation.

## Ethics Statement

The studies involving human participants were reviewed and approved by Ethics committee of Ulm University Application no: 188/15. Written informed consent to participate in this study was provided by the participants' legal guardian/next of kin.

## Author Contributions

SK, OW, and JS: conceptualization. KK and SK: methodology, data curation, and writing—original draft preparation. KK: software, formal analysis, and visualization. SK, OP, and KK: validation. SK and OW: investigation. JS: resources and funding acquisition. KK, OW, OP, JS, and SK: writing—review and editing. OP and SK: supervision. SK: project administration. All authors have read and agreed to the published version of the manuscript.

## Conflict of Interest

The authors declare that the research was conducted in the absence of any commercial or financial relationships that could be construed as a potential conflict of interest.

## Publisher's Note

All claims expressed in this article are solely those of the authors and do not necessarily represent those of their affiliated organizations, or those of the publisher, the editors and the reviewers. Any product that may be evaluated in this article, or claim that may be made by its manufacturer, is not guaranteed or endorsed by the publisher.
